# Flavone synthases from *Lonicera japonica* and *L. macranthoides* reveal differential flavone accumulation

**DOI:** 10.1038/srep19245

**Published:** 2016-01-12

**Authors:** Jie Wu, Xiao-Chen Wang, Yang Liu, Hui Du, Qing-Yan Shu, Shang Su, Li-Jin Wang, Shan-Shan Li, Liang-Sheng Wang

**Affiliations:** 1 Key Laboratory of Plant Resources/Beijing Botanical Garden, Institute of Botany, Chinese Academy of Sciences, Beijing 100093, China; 2 University of Chinese Academy of Sciences, Beijing 100049, China; 3 Key Laboratory of Plant Molecular Physiology, Institute of Botany, Chinese Academy of Sciences, Beijing 100093, China

## Abstract

Flavones are important secondary metabolites found in many plants. In *Lonicera* species, flavones contribute both physiological and pharmaceutical properties. However, flavone synthase (FNS), the key enzyme responsible for flavone biosynthesis, has not yet been characterized in *Lonicera* species. In this study, *FNSII* genes were identified from *Lonicera japonica* Thunb. and *L. macranthoides* Hand.-Mazz. In the presence of NADPH, the recombinant cytochrome P450 proteins encoded by *LjFNSII-1.1*, *LjFNSII-2.1*, and *LmFNSII-1.1* converted eriodictyol, naringenin, and liquiritigenin to the corresponding flavones directly. The different catalytic properties between LjFNSII-2.1 and LjFNSII-1.1 were caused by a single amino acid substitution at position 242 (glutamic acid to lysine). A methionine at position 206 and a leucine at position 381 contributed considerably to the high catalytic activity of LjFNSII-1.1. In addition, LjFNSII-1.1&2.1 and LmFNSII-1.1 also biosynthesize flavones that were further modified by *O*-glycosylation in transgenic tobacco. The expression levels of the *FNSII* genes were consistent with flavone accumulation patterns in flower buds. Our findings suggested that the weak catalytic activity of LmFNSII-1.1 and the relatively low expression of *LmFNSII-1.1* in flowers might be responsible for the low levels of flavone accumulation in flower buds of *L. macranthoides*.

Flos Lonicerae, commonly known as ‘*Jin Yin Hua*’, is derived from the flower buds of several *Lonicera* (honeysuckle) species that has been widely used in traditional Chinese medicine for thousands of years^1^,^2^. In China, more than 500 prescriptions used in the treatment of various diseases contain ‘*Jin Yin Hua*’^2^. They are mainly used to prevent and to treat hand-foot-and-mouth disease, H1N1 influenza, and severe acute respiratory syndromes^2^,^3^. ‘*Jin Yin Hua*’ is also an important and indispensable ingredient in some beverages^2^. Among these species, *Lonicera macranthoides* Hand.-Mazz. is the main species in south China^1^ and is usually used as a substitute for *L. japonica* Thunb, which has traditionally been the only natural plant source of ‘*Jin Yin Hua*’. Unlike *L. japonica*, which blossoms sporadically (Supplementary Fig. S1a), the flower buds of each inflorescence of *L. macranthoides* are prolific (Supplementary Fig. S1b). This trait makes *L. macranthoides* suitable for convenient and efficient harvesting. *L. macranthoides* has been grouped into the *Flos Lonicerae* (‘*Shan Yin Hua*’) instead of the *Flos Lonicerae japonica* (‘*Jin Yin Hua*’) by the China Pharmacopoeia since 2005. Although the two species are recorded in the China Pharmacopoeia, their bioactive compounds and medicinal usages are different. According to the China Pharmacopoeia, both luteolin-7-*O*-glucoside (Lu-7-Glc) and chlorogenic acids (CGAs) are standard compounds used for evaluating the chemical quality of *L. japonica*. However, only CGAs are used to evaluate the quality of *L. macranthoides*^4^. Many reports have linked flavones from the flower buds of *L. japonica* to a range of potential health benefits, such as anti-virus and anti-avian influenza virus activities^5^,^6^. Luteolin and Lu-7-Glc, the main flavones of *L. japonica*, are less abundant in buds of *L. macranthoides* than in *L. japonica*^1^,^3^. Therefore, understanding the molecular biology of flavone biosynthesis in these two *Lonicera* species should provide insight into potential metabolic engineering strategies for *L. macranthoides*, leading to increased biosynthesis and accumulation of these health-beneficial flavones.

Flavones are ubiquitous secondary metabolites in plants and are one of the largest subgroups of flavonoids^7^. Flavones are involved in various interactions with microbes, insects, and other plants^8^,^9^,^10^. In addition to their extensive functions in the biochemistry and physiology of plants, flavones are also important compounds for human nutrition and health^7^,^11^. Their medicinal properties, such as antioxidant, antiviral, anti-inflammatory activities and potential, have made these compounds increasingly popular as dietary constituents or supplements^7^. Therefore, these compounds are now understood to be attractive targets for metabolic engineering in plants and other organisms. Flavone biosynthesis is initiated by chalcone synthase, followed by chalcone isomerase^12^, which produces flavanones as precursors for the later synthesis of both flavones and other major flavonoid classes. The key enzyme responsible for the conversion of flavanones to flavones is flavone synthase (FNS); this enzyme catalyzes a double bond formation between C2 and C3 of flavanones (Fig. 1). Two distinct enzyme systems, specified by either FNSI or FNSII, have been shown to catalyze these reactions. The FNSI class consists of soluble Fe^2+^/2-oxoglutarate-dependent dioxygenases mainly described in members of the *Apiaceae* family^13^. FNSI enzyme has also been found in several monocotyledonous plants^14^,^15^,^16^ and in *Arabidopsis thaliana*^16^ in recent reports. The FNSII enzymes are NADPH-dependent cytochrome P450 membrane-bound monooxygenases widespread among many families of higher plants^7^. All characterized dicot FNSII enzymes belong to the CYP93B subfamily, but their catalytic mechanisms are different^17^. When they are expressed in heterologous systems, most FNSII enzymes exhibit a similar reaction mechanism with FNSI enzymes: converting flavanones directly to flavones. This reaction leading to flavones is involved in the synthesis of flavone *O*-glycosides (Fig. 1). During this conversion, two vicinal hydrogen atoms are abstracted to introduce a double bond in a radical-type mechanism. Interestingly, GeFNSII (CYP93B1) from the legume licorice (*Glycyrrhiza echinata*) and two MtFNSII enzymes (CYP93B10 and CYP93B11) from *Medicago truncatula* have flavanone 2-hydroxylation activity^18^,^19^ and are known to form a 2-hydroxyflavanone intermediate. In addition, flavone synthases from rice (CYP92G2), sorghum (CYP93G3), and maize (CYP93G5) have also been shown to be flavanone 2-hydroxylases, producing substrates for flavone *C*-glycosides biosynthesis *in planta*^12^,^20^,^21^,^22^.

Despite the important roles of flavones in *L. japonica* and *L. macranthoides*, the key enzymes for flavone biosynthesis have not yet been characterized in these species. Here, we report the functional characterization of FNSII enzymes from *L. japonica* and *L. macranthoides.* We identified three bona fide FNSII enzymes, and our results suggest that all three of these FNSII enzymes have a catalytic mechanism similar to most of the previously-identified FNSII enzymes, which exhibit a direct formation of flavones from flavanone substrates. In addition, these three FNSII enzymes generate flavones that are further modified by *O*-glycosylation in transgenic tobacco. Our results also demonstrate that there were differences in the catalytic activities between LjFNSII-1.1 and LmFNSII-1.1 that were caused by the site-directed substitution of amino acid sequences. In conclusion, our work assigns three genes as the first biochemically characterized enzymes that are involved in the biosynthesis of flavones and flavone *O*-glycosides in *L. japonica* and *L. macranthoides*. The catalytic differences and the differences in the expression patterns of *FNSII*s likely result in the differential flavone accumulation between *L. japonica* and *L. macranthoides.*

## Results

### Isolation of the candidate *FNS* genes from *L. japonica* and *L. macranthoides*

**Figure 2:**
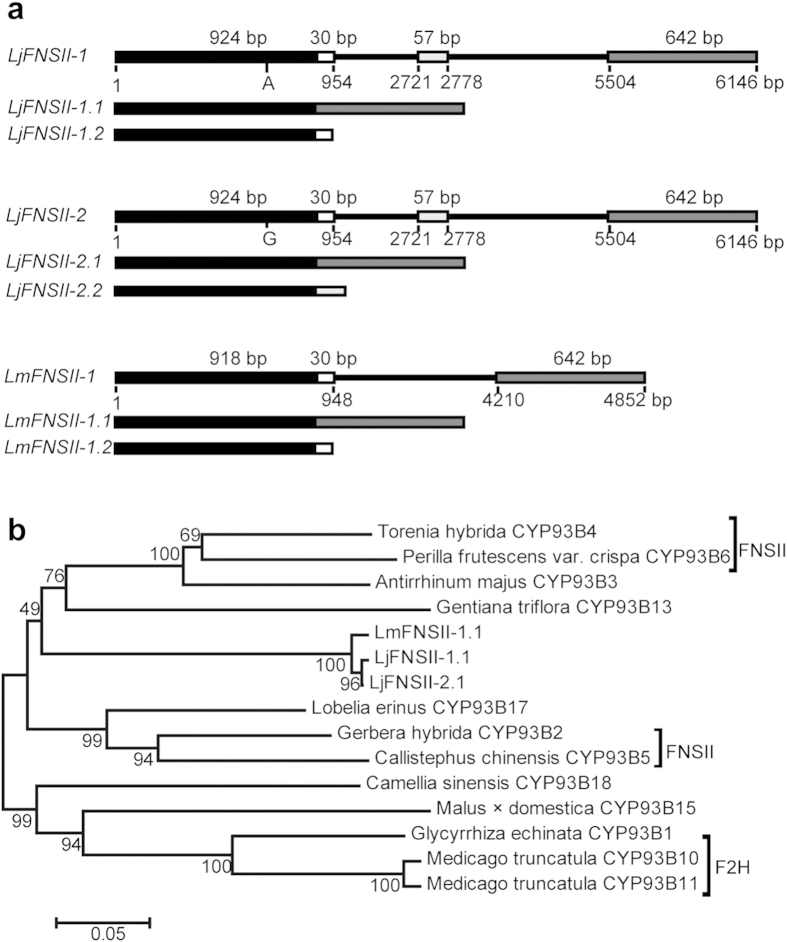
Schematic diagram of the *LjFNSII-1&2* and *LmFNSII-1* genes and phylogeny analysis. (**a**) The upper drawing shows the gene structures and the alternative transcript forms of the *FNSII* genes from *Lonicera japonica* (*LjFNSII-1&2*) and *L. macranthoid*es (*LmFNSII-1*). *LjFNSII-1* (KU127584) and *LjFNSII-2* (KU127585) have similar genomic structures and a small number of polymorphisms. However, they display different splicing patterns. Only one polymorphism between their base sequences (A to G) causes an amino acid substitution between *LjFNSII-1* and *LjFNSII-2*. The intron sequence of *LmFNSII-1* (KU127586) is highly divergent from *LjFNSII-1*, though they exhibit similar splicing patterns. The diagram shows the exons (solid black, white, and grey boxes) and introns (black lines) of the *FNSII* genes. The numbers represent the lengths of the exons and introns. Each *FNSII* gene encodes two different transcripts. (**b**) Phylogeny analysis among selected FNSII family members. The FNSII proteins were grouped into two clades based on sequence homology and catalytic specificity. The unrooted tree was constructed with a Neighbor-Joining phylogeny analysis using MEGA 4.0. Numbers at the nodes represent bootstrap values from 1,000 replicates. The accession numbers of all genes used for the unrooted phylogenetic tree are shown in Supplementary Table S2.

RNA-seq analysis of *L. japonica* provided a partial cDNA sequence for an *FNS* gene (JX068613)^3^. Based on this information, similar sequences were separately amplified by reverse transcription PCR from *L. japonica* and *L. macranthoides*, and the full-length cDNA sequences were then recovered by 5′ and 3′ RACE PCR. In total, these experiments led to the identification of six putative *FNSII*s that were designated as *LjFNSII-1.1* (KU127576), *LjFNSII-1.2* (KU127577), *LjFNSII-2.1* (KU127578), *LjFNSII-2.2* (KU127579), *LmFNSII-1.1* (KU127580), and *LmFNSII-1.2* (KU127581) (Supplementary Table S1). The DNA sequences of *LjFNSII-1* indicated that *LjFNSII-1.1* and *LjFNSII-1.2* were products of alternative splicing at two sites, which was true for the other two pairs of alternative splice variants (Fig. 2a). A sequence identical to *LjFNSII-2.2* was also found in *L. macranthoides* (Supplementary Fig. S1c).

**Figure 3:**
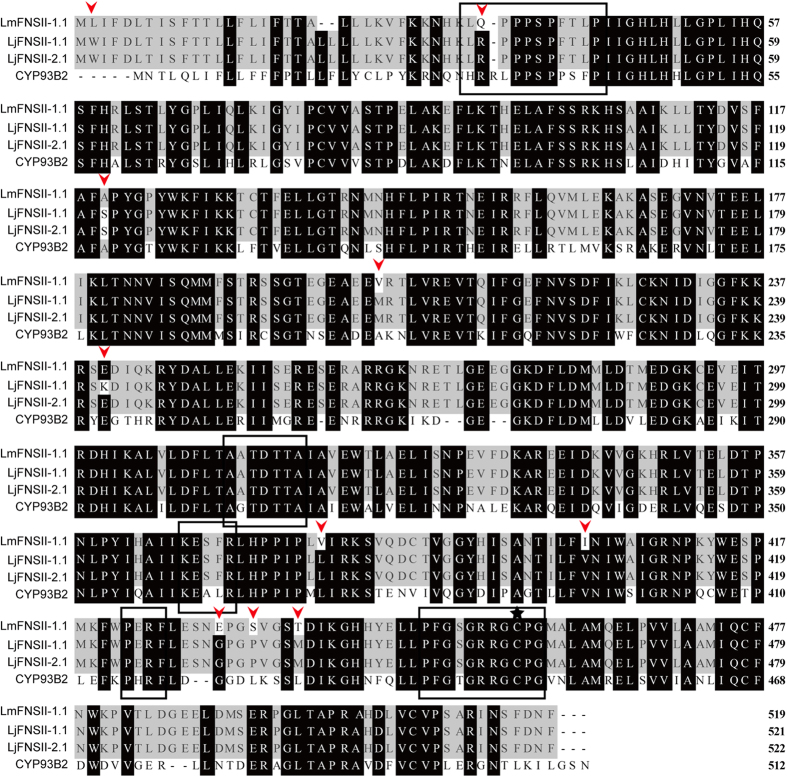
Comparison of *Lonicera* FNSII proteins with other FNSII proteins. The amino acid sequences of FNSII from *L. japonica* (LjFNSII-1.1&2.1) and *L. macranthoid*es (LmFNSII-1.1) were aligned with the amino acid sequence of GhFNSII from *Gerbera hybrida* (CYP93B2) using MEGA 4.0. The proline rich membrane hinge (LQ/RPPPSP), I-helix (AATDTTA), E-R-R triade consisting of the K-helix consensus sequence (KESFR) and the consensus sequence (PERF), heme-binding domain (PFGSGRRGCPG) are boxed; these are conserved motifs among P450s. The cysteine in the heme-binding domain is marked by black stars and is conserved in all plant P450 sequences. Residues shaded in black indicate identical amino acids, and different residues from LjFNSII-1.1&2.1 and LmFNSII-1.1 are indicated by arrows.

Comparison of deduced amino acid sequences revealed that the putative LjFNSII-1.1, LjFNSII-2.1, and LmFNSII-1.1 proteins harbored the characteristic sequence signatures of the cytochrome P450 family, including a Pro hinge region, a heme-binding motif, and an oxygen-binding pocket^23^ (Fig. 3). However, LjFNSII-1.2, LjFNSII-2.2, and LmFNSII-1.2 were shorter, and lacked typical domains, including oxygen-binding pockets and catalytic sites, suggesting that these are not functional cytochrome P450 proteins. LjFNSII-1.1 and LjFNSII-2.1 only had a single nucleotide polymorphism at position 242 (this polymorphism encoded a Lys to Glu substitution). There were 10 different amino acids between the sequences of LjFNSII-1.1 and LmFNSII-1.1; they shared, respectively, 62.10% and 62.07% identity to GhFNSII (CYP93B2)^24^ at the amino acid level (Fig. 3). A neighbor-joining phylogenetic tree was constructed using the deduced amino acid sequences of LjFNSII-1.1, LjFNSII-2.1, and LmFNSII-1.1, and other flavone synthase II sequences from other plants, including some known to be involved in flavanone 2-hydroxylation activity (Fig. 2b, Supplementary Table S2). These FNSIIs were grouped into two major families. LjFNSII-1.1, LjFNSII-2.1, and LmFNSII-1.1 were grouped into the same clade as CYP93B3, CYP93B4, CYP93B6, and CYP93B13, and these were clearly separated from the flavone synthases involved in 2-hydroxyflavanone biosynthesis. These results suggested that the putative FNSII proteins from *L. japonica* and *L. macranthoides* belonged to the FNSII clade, the enzymes of which are known to putatively function in the production of flavones directly from flavanones.

### *In vivo* yeast expression assays of the LjFNSII and LmFNSII proteins

To investigate the catalytic activity of the enzymes encoded by the isolated putative *FNSII* genes, the coding region of each sequence was inserted into the pYeDP60 vector^25^ and transformed into the WAT11 yeast strain, which expresses the *Arabidopsis* NADPH-cytochrome P450 reductase^26^ and provides the reducing equivalents indispensable for the activity of plant CYP450s such as flavone synthase II enzymes.

**Figure 4:**
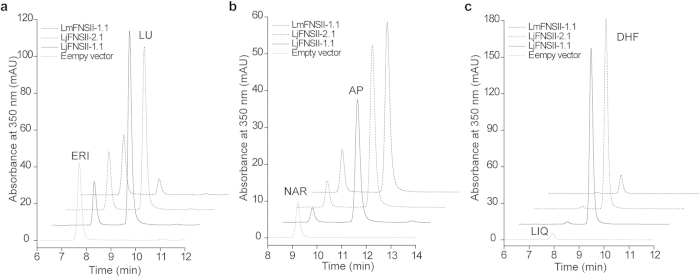
HPLC profiles of extracts from yeast cells expressing LjFNSII-1.1&2.1 and LmFNSII-1.1. 350 nm UV spectra for HPLC chromatograms show the direct conversion of (**a**) eriodictyol (ERI) to luteolin (LU), (**b**) naringenin (NAR) to apigenin (AP), and (**c**) liquiritigenin (LIQ) to and 7, 4′-dihydroxyflavone (DHF), respectively. The yeast cells transformed with the empty vector were used as the negative control. Dotted lines, reactions incubated with the empty vector; Solid black lines, reactions incubated in the presence of LjFNSII-1.1; Dashed lines, reactions incubated in the presence of LjFNSII-2.1; Dashed-dotted lines, reactions incubated in the presence of LmFNSII-1.1.

Transformed yeast cells expressing LjFNSII-1.1, LjFNSII-2.1, and LmFNSII-1.1 were found to metabolize eriodictyol, naringenin, and liquiritigenin to luteolin, apigenin, and 7, 4′-dihydroxyflavone (DHF), respectively (Fig. 4). The control cells carrying the empty vector did not generate any detectable luteolin, apigenin or DHF (Fig. 4), nor did the cells expressing the short *FNSII* genes (Supplementary Fig. S2a). The identification of luteolin, apigenin, and DHF produced by the transformed yeast cells was confirmed by comparison of the retention times, the UV absorption spectra, and the mass fragmentation patterns with those of authentic standards (Supplementary Fig. S2b). There were no other intermediate compounds detected, as had been the case with heterologously expressed FNSII proteins from *M. truncatula*^19^. This analysis indicated that LjFNSII-1.1, LjFNSII-2.1 and LmFNSII-1.1 are functional FNSIIs and that they directly convert flavanones into corresponding flavones, while shorter FNSIIs might lose their function of flavone synthesis.

### *In vitro* enzyme activity assays of recombinant FNSII proteins

To investigate the enzymatic properties of LjFNSII and LmFNSII proteins, the microsomes were prepared from transformed yeast cells. Microsomal suspensions were incubated with flavanone substrates in potassium phosphate buffer in the presence of NADPH, and the extracts of the reaction mixtures were analyzed by HPLC.

The activities of microsomal extracts containing LjFNSII-1.1, LjFNSII-2.1, and LmFNSII-1.1 were strongly affected by changes in pH and changes in temperature. The highest activities were observed under nearly neutral pH around 7.5; activities were dramatically reduced when more acidic conditions were tested (Supplementary Fig. S3a). The enzymes exhibited their highest catalytic activities at 37 °C, and they were rapidly denatured at higher temperatures (Supplementary Fig. S3b). The activities of LjFNSII-1.1, LjFNSII-2.1, and LmFNSII-1.1 were affected by the presence of divalent metal cations. Their activities were found to decrease in buffer containing Ca^2+^, Mn^2+^, Ni^2+^, Co^2+^, or Zn^2+^, and the presence of Cu^2+^or Fe^2+^ in the buffer prevented the production of flavones (Supplementary Fig. S4).

Under these optimized conditions, the catalytic efficiencies of LjFNSII-1.1, LjFNSII-2.1, and LmFNSII-1.1 for three different flavanones (eriodictyol, naringenin, and liquiritigenin) were determined by quantifying the production of their corresponding flavones. Each of the enzymes utilized the substrates with affinities in the low apparent *K*_*m*_ range (Table 1). The apparent catalytic efficiency of LjFNSII-1.1 with flavanone substrates was considerably higher than that of LjFNSII-2.1 or LmFNSII-1.1. Based on a comparison of the *K*_*cat*_*/K*_*m*_ values, the FNSII enzymes exhibited a marked preference for liquiritigenin and eriodictyol over naringenin. Although LjFNSII-1.1 and LmFNSII-1.1 showed similar substrate specificity and biochemical properties, LjFNSII-1.1 exhibited an approximate 4-fold higher activity than that of LmFNSII-1.1 when eriodictyol was used as the substrate.

### Amino acid substitution results in differences among the catalytic activities of the FNSII enzymes

Considering that there is only a single amino acid substitution between LjFNSII-1.1 and LjFNSII-2.1, it is reasonable to assume that the residue Glu-242 to Lys change might not only increase the overall activity but also increase the apparent *K*_*m*_ (Table 1). The site-directed mutant LmFNSII-1.1-E240K also exhibited similar behavior, with elevated catalytic rates and increased apparent *K*_*m*_ as compared to LmFNSII-1.1 (Supplementary Table S3). When the residue Glu242 was mutated into a neutral glycine (LjFNSII-2.1-E242G), the reaction rate increased and the apparent *K*_*m*_ increased slightly compared to LjFNSII-2.1 (Supplementary Table S3). These results indicated that a basic amino acid at position 242 led to faster catalytic reactions than an acidic or a neutral one.

For each substrate, the catalytic efficiency of LjFNSII-1.1 was much higher than that of LmFNSII-1.1. The high amino acid sequence identities between them indicated that their divergent enzymatic activities likely resulted from differences in amino acid residues. Several site-directed mutants were constructed to investigate key active-site residues of these FNSII enzymes. When assayed under the optimized reaction conditions with eriodictyol as the substrate, the observed *in vitro* catalytic efficiency of each mutant was evaluated. Among the mutated enzymes, LmFNSII-1.1-V204M displayed 222.68% of the activity of LjFNSII-1.1 (100%) (Table 2). By contrast, the activity of LmFNSII-1.1 was only 21.17% of the activity of LjFNSII-1.1. These results indicated that a methionine at position 206 contribute substantially to the high catalytic activity of LjFNSII-1.1. On the other hand, LmFNSII-1.1-V379L displayed 130.61% of the activity of LjFNSII-1.1 (Table 2); other LmFNSII-1.1 mutants showed less substantial or inconspicuous increases in activity as compared with LmFNSII-1.1 (data not show). To validate whether Met-206 and Leu-381 are key residues in the active-sites, two mutations (M206V and L381V) of LjFNSII-1.1 were constructed. Both of the mutants showed dramatic decreases in catalytic activity compared to that of LjFNSII-1.1(Table 2). Kinetic studies showed that the catalytic efficiency of LmFNSII-1.1-V204M was significantly increased as compared to LmFNSII-1.1, to nearly the same level as that of LjFNSII-1.1 (Supplementary Table S3). These results indicated that both Met-206 and Leu-381 were critical residues for the catalytic activity of LjFNSII-1.1.

For LmFNSII-1.1-A120S, there was hardly any detectable product; its activity was only 0.35% of that of LjFNSII-1.1 (100%). Kinetic studies of LjFNSII-1.1-S122A showed that there was a dramatic increase in the catalytic rate compared to LjFNSII-1.1 (Supplementary Table S3). This indicated that the alanine at this position was critical for the catalytic activity of the FNSII enzymes and was indispensable for the catalytic activity of LmFNSII-1.1.

### Structural insights from homology modeling and docking

**Figure 5:**
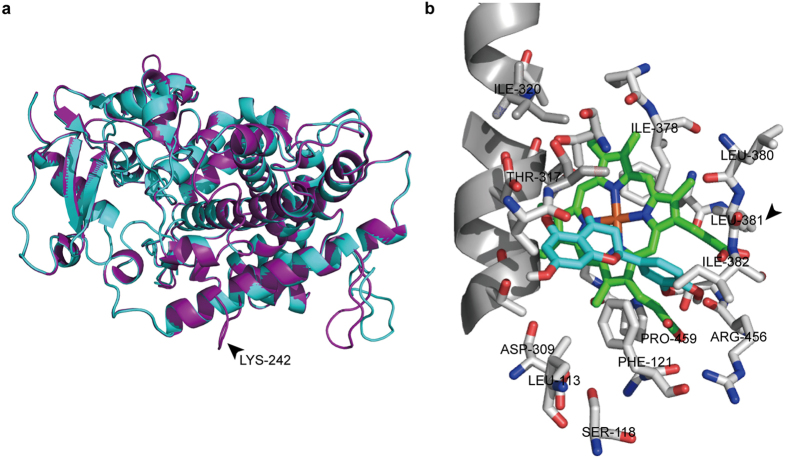
Homology modeling and molecular model of the active site of LjFNSII-1.1. (**a**) Alignment of models of LjFNSII-1.1 and LjFNSII-2.1. The structure of an α-helix of LjFNSII-1.1 was interrupted by the presence of the Lys-242 residue. The secondary structures of LjFNSII-1.1 and LjFNSII-2.1 are marked in purple and cyan, respectively. (**b**) The eriodictyol and the heme were docked in the generated model of LFNSII-1.1. The eriodictyol and heme are labeled in cyan and green, respectively. The substrate-binding residues are represented as sticks. In this model, the carbonyl oxygen atoms of the residues Arg-456 were involved in putative hydrogen bonds with the hydroxyl groups of eriodictyol, which may help to bind the substrate in its correct orientation towards the enzyme. Thr-317 and Ile-378 are likely critical for correct substrate positioning as well, favoring the abstraction of hydrogen from C-2 rather than from C-3 so as to synthesize flavones. Ile-382 was predicted to be involved in the maintenance of the P450 conformation through hydrogen bond interactions with the heme. The heme cofactor was kinked in a bent conformation due to close interaction with a proline residue (Pro-459).

Homology modeling and docking with the substrate were performed to provide insights into the structural features and basis for differences in catalytic activities among LjFNSII-1.1, LjFNSII-2.1, and LmFNSII-1.1. Three-dimensional homology models of LjFNSII-1.1 and LjFNSII-2.1 were built based on the structures of CYP1A1 (30.13% identity and 29.91% identity, respectively). Alignment of the LjFNSII-1.1 and LjFNSII-2.1 models revealed that one α-helix of LjFNSII-1.1 was interrupted due to the presence of Lys-242 (Fig. 5a). *In silico* docking analysis of the eriodictyol and the heme moiety in the generated protein model of LjFNSII-1.1 showed that several residues were predicted to be part of the putative substrate-binding pocket of LjFNSII-1.1, including Phe-121, Thr-317, Ile-378, Leu-380, Leu-381, Ile-382, Arg-456, Pro-459 (Fig. 5b). In the structure model, the residues Thr-317, Ile-378^27^, and Pro-459 were conserved, and appeared to function in similar ways as those of other P450 proteins^28^,^29^. In addition, the Leu-381 and the positively-charged Arg-456 were found to be located immediately above the heme plane and appear to dominate the local structure, a situation that may influence the enzymatic catalysis. However, Met-206, which contributed substantially to the catalytic activity of LjFNSII-1.1, was far from the putative pocket.

### *In planta* characterization of FNSII activities

**Figure 6:**
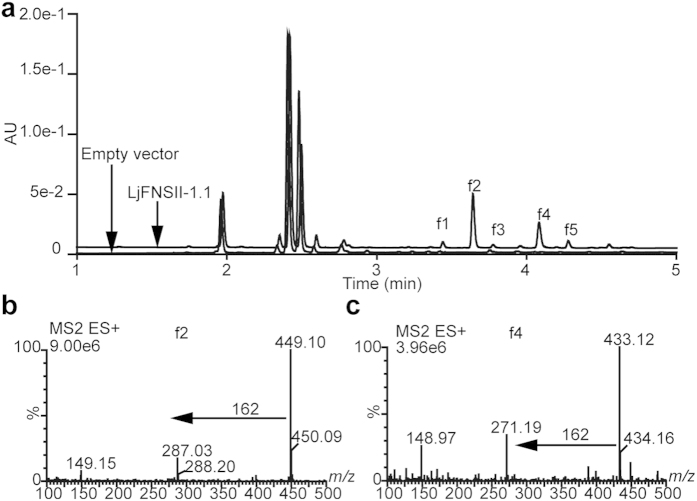
Characterization of FNS activity *in vivo* by transforming *N. benthamiana*. (**a**) Flavone profiles of transgenic leaves (*35S::LjFNSII-1.1* and empty vector control, respectively). (**b**) The MS/MS fragmentation patterns of peak f2. (**c**) The MS/MS fragmentation pattern of f4.

To understand their metabolic roles *in planta*, the coding sequences of *LjFNSII*-*1.1*, *LjFNSII-2.1*, and *LmFNSII-1.1* were placed under the control of the cauliflower mosaic virus 35S promoter and these constructs were introduced via *Agrobacterium*-mediated transformation into *N. benthamiana* to investigate their activities *in vivo*. Transgenic tobacco lines were found to accumulate the *FNSII* transcripts, and their flavone compositions were analyzed by UPLC-MS (Fig. 6a). Several compounds accumulated in tobacco leaves, and the metabolites were putatively identified from their elution order, UV absorption spectra, and mass fragmentation patterns. Due to the labile nature of the *O*-glycosidic linkages, fragmentations leading to the loss of sugar moieties are easily induced during MS/MS experiments. Hence the peaks (f1–f5) were putatively identified as *O*-hexoside compounds (Supplementary Table S4 and Supplementary Fig. S5).

The most prominent peak, f2 (*m/z* 449 [M + H]^+^), generated the diagnostic ion *m/z* 287 of luteolin during MS/MS fragmentation (Fig. 6b). The loss of 162 D is indicative of the presence of an *O*-linked hexose in the parent compound. Similarly, the other prominent peak was identified as the *O*-hexoside (f4, *m/z* 433 [M + H]^+^) of apigenin (*m/z* 271) (Fig. 6c). The two peaks were identified by comparison with authentic reference standards and assigned as luteolin-7-*O*-glucoside (f2, Lu-7-*O*-Glc) and apigenin-7-*O*-glucoside (f4, Ap-7-*O*-Glc), respectively. These two flavones accounted for most of the accumulated flavones content in the transgenic leaves. Peaks f1, f3, and f5 were found to accumulate slightly in some transgenic lines. The total flavones were found to accumulate differentially in the transgenic lines expressing LjFNSII-1.1, LjFNSII-2.1, and LmFNSII-1.1 (Supplementary Fig. S6). The flavones were accumulated as a result of the FNSII activities in the transgenic lines, and these compounds were further modified by the endogenous flavonoid *O*-glycosyltransferases. These results further validated the FNSII activities of LjFNSII-1.1, LjFNSII-2.1, and LmFNSII-1.1 *in planta*, which involved in the synthesis of flavones and flavone *O*-glycosides.

### The expression patterns of *FNSII* genes are consistent with flavone accumulation patterns

**Figure 7:**
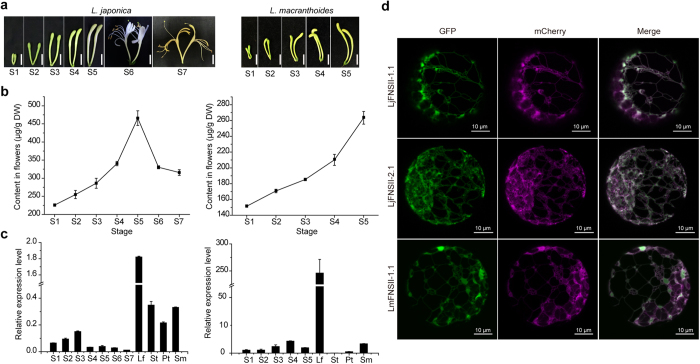
Flavone accumulation at different stages, expression patterns of *FNSII*s and sub-cellular location of FNSIIs proteins. (**a**) Flowers at seven stages (S1–S7) from *L. japonica* and five stages (S1–S5) from *L. macranthoides*. The absence of flowers of *L. macranthoides* at stages 6 and 7 was due to the fact that flower buds of *L. macranthoides* keep closed during the whole flower development stage. Scale bars = 1.0 cm. (**b**) Flavone accumulation at different stages. (**c**) Relative expression levels of *LjFNSII-1.1&2.1* and *LmFNSII-1.1* at the corresponding stages and in different tissues, including leaf (Lf), stem (St), petal (Pt), and stamen (Sm). Expression values have been normalized with the actin gene. The graph shows average values of three replicates with the respective error bars indicative of the standard deviation. (**d**) The transient expression of FNSII-GFP fusions in *Arabidopsis* protoplasts. Transient expression constructs (*35S-FNSII-GFP* and *35S-mCherry-HDEL*) were co-transformed into *Arabidopsis* protoplasts. Merge and overlay of FNSII-GFP fluorescence and mCherry-HDEL fluorescence (endoplasmic reticulum [ER] marker). Scale bars = 10 μm.

To investigate whether there was any correlation between the expression levels of the *FNSII* genes and flavone accumulation levels during the floral development of *L. japonica* and *L. macranthoides*, flower buds at different development stages were harvested and analyzed for both gene expression and flavone accumulation. The total expression level of the *LjFNSII*-*1.1* and *LjFNSII*-*2.1* in *L. japonica* was evaluated because of the high degree of identity between their sequences. As shown in Fig. 7a–c, the expression level of *LjFNSII*-*1.1&2.1* continuously increased and reached a maximum at stage S3, while the flavones in flowers continued to increase until S5, likely due to the hysteresis effect of gene transcripts. The flavone content decreased after blooming. In contrast, the expression level of *LmFNSII-1.1* in *L. macranthoides* was highest at stage S4, while flavones continued to accumulate because the flower buds of *L. macranthoides* were unable to bloom. The results indicated that *FNSII* expression levels were consistent with the flavone accumulation patterns in flowers buds.

In addition, *FNSII* expression was also evaluated in leaves, stems, flower petals, and stamens. *LjFNSII-1.1&2.1* transcripts were more highly expressed in leaves of *L. japonica* as compared with that in flower buds or stems. These results confirmed that flavone contents were highest in leaves of *L. japonica*, but much lower in flowers and stems^2^,^3^. The expression level of *LmFNSII-1.1* in leaves of *L. macranthoides* was about 20 times higher than that in flower buds of *L. japonica*, leading up to as high accumulation of flavones in the leaves of *L. macranthoides* as that of *L. japonica* (Supplementary Table S5). Moreover, a bacterial uidA (GUS) reporter cassette was placed under the control of the *LmFNSII-1.1* promoter and their tissue-specific expression patterns were examined in transgenic *N. benthamiana* with histochemical staining. This experimental system was chosen because of the lack of a successful genetic transformation system for use in *Lonicera* species (Supplementary Fig. S7). The strongest GUS staining was observed in leaves and stamens. These results confirmed the low expression levels of *LmFNSII-1.1* in flowers petals of *L. macranthoides*, which suggested that the low catalytic efficiency of the LmFNSII-1.1 enzyme and the unique expression pattern of *LmFNSII*-*1.1* might cause the low content of flavones in the flower buds of *L. macranthoides*.

### Subcellular localization of the LjFNSII and LmFNSII proteins

All P450s reported so far in plants are membrane-localized^30^. The FNSII proteins from *L. japonica* and *L. macranthoides* all have a predicted signal peptide at their N-terminus. To investigate whether they are targeted to the endoplasmic reticulum or to other cellular locations, the subcellular localizations of the FNSII proteins were observed by transiently co-expressing C-terminal GFP fusion constructs with the ER marker (mCherry-HDEL) in *Arabidopsis* Col-0 protoplasts. As shown in Fig. 7d, the FNSII-GFP fusion fluorescence was merged to the ER marker in protoplasts, suggesting that the FNSII proteins were anchored on the endoplasmic reticulum. The shorter transcripts of *LjFNSII-1* and *LjFNSII-2* formed by alternative splicing modes of the introns were also likely anchored on the endoplasmic reticulum (Supplementary Fig. S8). They exhibited the same localization signals as the longer transcripts, on account of the identical hydrophobic peptides present at the N-terminus of the proteins they encode. These results demonstrated that all of the FNSII proteins identified from *L. japonica* and *L. macranthoides* were targeted to the endoplasmic reticulum in this experimental system.

## Discussion

Flavones have diverse functions *in planta*. Their important roles include alteration of the color of flowers and leaves with anthocyanins, UV protection, and their functions as reservoirs of phytoalexins^31^,^32^. In addition to their important physiological roles, flavones have been demonstrated to exhibit significant pharmacological activities in mammals. For example, luteolin has been shown to induce apoptosis, inhibit angiogenesis, and to have antioxidant and anti-inflammatory activities, as well as strong cytotoxicity against tumor cells^33^,^34^. The flavones from flower buds of *L. japonica*, especially for luteolin and Lu-7-Glc, were found to have various medicinal effects^2^,^5^. However, luteolin and Lu-7-Glc are only accumulated at low levels in *L. macranthoides*, a plant that is used as a substitute for *L. japonica* in traditional medicine in many places. To better understand the multiple roles of flavones and the basis for the diverse flavone content in *L. japonica* and *L. macranthoides*, we identified three functional *FNSII* genes from *L. japonica* and *L. macranthoides* and investigated their biochemical properties *in vitro* and studied their functions *in vivo*.

We isolated two genes from *L. japonica*; both of them displayed two different splicing variants. The shorter splicing variants of *LjFNSII-1* and *LjFNSII-2* did not, when expressed in yeast, lead to flavone accumulation, suggesting that the shorter splicing variant transcripts did not code for functional FNSII enzymes. This is likely, given the absence of the oxygen-binding and heme-binding domains at the C-terminus. Their other functions of shorter transcripts of *LjFNSII-1* and *LjFNSII-2 in planta* remain as yet undiscovered. However, the intron position and phase of P450s are considered as fossils of the evolutionary relationship^35^, and they might be used as diagnostic fingerprints for validating the phylogeny of *Lonicera* species. The counterpart of *LjFNSII-2.2* was found in *L. macranthoides* while that of *LjFNSII-2.1* was not detected in our RT-PCR experiments. This suggested that there were two *FNSII* genes in *L. macranthoides* and that one of them might have become non-functional following gene duplication. It has been shown that the CYP71 clan in angiosperms, which consists of CYP93B and other subfamilies, has evolved by intensive gene duplication^36^; the mechanism for selectively retaining some of these duplicated genes as functional enzymes is unknown.

LjFNSII-1.1 and LjFNSII-2.1 were found to have only a single amino acid difference between them. The substitution of an acidic amino acid (Glu-242) of LjFNSII-2.1 for a basic amino acid (Lys-242) in LjFNSII-1.1 contributed to the α-helix stability (Fig. 5a). How the α-helix weakens the catalytic efficacy is unknown. Nevertheless, it is worth exploring how these two FNSII enzymes inter-coordinate with each other in *L. japonica*. As is often seen, genes that have undergone neo- or sub-functionalization are sometimes differentially regulated developmentally and spatially^36^. A well-documented example of this was found in *M. truncatula* where there were two FNSII homologs displaying distinct tissue-specific expression patterns. MtFNSII-2, predominantly expressed in flowers, was induced by defense or symbiotic signals and highly expressed in roots^19^. This result implied coordinate activation of enzymes upon the reception of defense signals^37^ and different roles of flavones in UV protection (in flowers) and as signal molecules (in roots). We were unable to ascertain the respective expression patterns of *LjFNSII-1.1* and *LjFNSII-2.1* by RT-PCR due to the high degree of similarity between their sequences. However, their promoters are quite distinct (Supplementary Fig. S9), indicating that they may have disparate expression patterns and may very well be regulated by different factors. Further investigation is necessary to understand what role these two FNSII homologs from *L. japonica* play and how they might coordinate with each other.

Our results confirmed that the recombinant FNSII enzymes from *L. japonica* and *L. macranthoides* showed low *K*_*m*_ values for flavanones (Table 1). These values in the low molar concentration range are similar to those of the previously characterized recombinant FNSII enzymes. For instance, determination of the kinetic parameters for a recombinant FNSII enzyme from *Perilla frutescens* (CYP93B6) revealed that the *K*_*m*_ values for naringenin and eriodictyol were 8.8 μM and 11.9 μM, respectively^38^. A recombinant FNSII enzyme from *Gentiana triflora* (CYP93B13) and one from soybean (CYP93B16) exhibited *K*_*m*_ values of 8.9 and 2.5 μM with naringenin, and 19.1 and 1.8 μM with eriodictyol, respectively^17^,^39^. In all of the cases described above, naringenin was more efficiently converted and was preferred as an *in vitro* substrate over eriodictyol. While a recombinant FNSII enzyme from rice (CYP93G1) exhibited *K*_*m*_ values of 3.2 and 1.5 μM with those two flavanone substrates, and had a relatively larger *V*_*max*_*/K*_*m*_ ratio for eriodictyol than for naringenin, which suggested that CYP93G1 converted eriodictyol more efficiently than it converted naringenin^40^. In *L. japonica* and *L. macranthoides*, the FNSII enzymes preferred eriodictyol as a substrate over naringenin as well. The *K*_*cat*_*/K*_*m*_ values of eriodictyol were about four times higher than those for naringenin for both LjFNSII-1.1 and LjFNSII-2.1. The results are consistent with the fact that luteolin and its derivates are the main flavones in *L. japonica*, rather than apigenin and its derivatives^1^. The difference in catalytic efficiency of LjFNSII-1.1 and LmFNSII-1.1 also implied relatively higher flavone content in *L. japonica* compared to *L. macranthoides*. Although the FNSII enzymes had high *K*_*cat*_*/K*_*m*_ values for liquiritigenin, a legume-specific flavanone, it is unlikely to be an *in vivo* substrate. The catalytic activities of FNSII enzymes against liquiritigenin are likely due to broad substrate preferences *in vitro*. Different activity profiles of enzymes *in vitro* and *in vivo* have also been described in the cases of several *UGT* genes from *A. thaliana*^41^,^42^ and *M. truncatula*^43^.

The difference in catalytic efficiency between LjFNSII-1.1 and LmFNSII-1.1 might be a consequence of the differences in their amino acid sequence. Site-directed mutation of these FNSII enzymes suggested that a methionine at position 206 and a leucine at position 381 were critical for the high catalytic activity of LjFNSII-1.1. To verify the role of these amino acids in these enzymes, 3D modeling of LjFNSII-1.1 was conducted. These conserved domains of P450s were identical between LjFNSII-1.1 and LmFNSII-1.1. However, the leucine at position 381 in LjFNSII-1.1 is adjacent to the K-helix that forms the E-R-R triad of P450s involved in locking the heme pockets into position and stabilizing the conserved structure^44^; the mutation might affect the stabilization of heme as compared with the corresponding Val-379 of LmFNSII-1.1, which removes a methyl group from the active-site. Met-206 in LjFNSII-1.1 was predicted to be far from the putative substrate-binding pocket; however, when we replaced the Met-206 with valine and performed enzymatic assays, the catalytic activity of LjFNSII-1.1 was decreased dramatically. This may be explained by the possibility that methionine was involved in the formation of a disulfide bridge, which is a prerequisite for the proper biological function of these proteins^45^. The effect of Ala-120 in LmFNSII-1.1 is difficult to explain. The replacement of Ala-120 in LmFNSII-1.1 with a hydrophilic threonine residue resulted in a dramatic decrease in catalytic activity. The threonine in LjFNSII-1.1 was predicted to be located next to the active-site Phe-121, as shown in Fig. 5b. The hydrophilic residue may cause protein instability or unfavorable substrate accommodation, considering the fact that active sites of CYPs are predominantly hydrophobic in nature^46^. Although many P450 crystal structures have now been characterized in mammals, fungi, and bacteria, no plant P450 structures have yet been solved^47^. Most plant P450s share low identity in amino acid sequence compared with the available crystallographic templates, which indicates that it might be difficult to generate exact P450 models for the target proteins^47^. Hence, the mechanism for the strong contribution of Met-206 and Leu-381 to the catalytic efficiency of LjFNSII-1.1, and that of Ala-120 to LmFNSII-1.1, should be investigated further.

In this study, we conclude that the weak catalytic activity of LmFNSII-1.1 might be responsible for the low level accumulation of flavones in buds of *L. macranthoides*, in spite of the relatively higher expression of *LmFNSII-1.1* in buds as compared to the expression levels of *LjFNSII-1.1* in buds of *L. japonica*. The high expression level of *LmFNSII-1.1* and the high level of flavone accumulation in leaves of *L. macranthoides* suggest that the lower level of flavones might be offset by the high expression level of the flavone synthase. The work also suggests that the flexibility of plant metabolism can allow the diversion of a substrate towards the biosynthesis of health-beneficial flavone derivatives. *L. macranthoides* may represent a good substitute for *L. japonica* if its flavone content can be elevated, considering the abundance of its flower buds. Therefore, the modification of the flavone biosynthesis pathway for the metabolic engineering of *L. macranthoides* to accumulate these health-beneficial flavones in *L. macranthoides* is worthy of further research.

## Methods

### Chemical sources

Naringenin (NAR), eriodictyol (ERI), liquiritigenin (LIQ), luteolin (LU), apigenin (AP), 7, 4′-dihydroxyflavone (DHF), and diosmetin (Dio) were purchased from Shanghai Tauto Biotech (China). Luteolin 7-*O*-glucoside (Lu-7-*O*-Glc) and apigenin 7-*O*-glucoside (Ap-7-*O*-Glc) were generously offered by Professor Xiao Wang (Shandong Analysis and Test Center, Shandong Academy of Sciences, China). Chromatographic grade methanol and acetonitrile were obtained from Alltech Scientific (USA), and other analytical regents were obtained from Beijing Chemical Works (China).

### Plant materials and culture conditions

*Lonicera macranthoides* was collected from the Hunan Xiangzhong Honeysuckle Technology Development Company (China). *L. japonica* was grown at Beijing Botanical Garden (Institute of Botany, Chinese Academy of Sciences, China). The *Arabidopsis thaliana* and *Nicotiana benthamiana* plants used in this study were cultivated in a greenhouse under 12-h light/12-h dark photoperiod for *A. thaliana* and a 16-h light/8-h dark for *N. benthamiana*. The temperature was maintained at 25 °C during the light phase and 22 °C during the dark phase.

### Cloning and phylogenetic analysis of *FNSII* genes from *L. japonica* and *L. macranthoides*

Total RNA from *L. japonica* and *L. macranthoides* was extracted from young flower buds and leaves using RNA-prep Pure Plant Kits (Tiangen, China). cDNA for the rapid-amplification of cDNA ends (RACE) was prepared according to the user manual for the SMART RACE cDNA Amplification Kit (Clontech, Japan), and the cDNA for the RT-PCR analyses was synthesized using the AMV Reverse Transcription System (Promega, USA). Genes were amplified by using a Fast HiFidelity PCR Kit (Tiangen, China), according to the manufacturer’s instructions; PCR products were cloned into the pLB vector (Tiangen, China) and sequenced. The sequences of newly found genes from this study were submitted to the GenBank database and the accession numbers are listed in Supplementary Table S1. For sequence alignment and phylogenetic analysis, the deduced amino acid sequences were compared with the MEGA 4.0 program using the Neighbor-Joining method with 1000 bootstrap replications^48^.

### Subcellular localization of the FNSII proteins

The coding sequence of each FNSII protein from *L. japonica* and *L. macranthoides*, lacking the stop codon, was inserted into the pBI-221 vector (Invitrogen, USA) to generate a C-terminal GFP fusion driven by the Cauliflower mosaic virus (CaMV) 35S promoter. Transient expression vectors were transformed into *Arabidopsis* Col-0 protoplasts as described (http://genetics.mgh.harvard.edu/sheenweb/). The GFP fusions were co-expressed with an ER marker fusion (mCherry–HDEL) that was generated by linking the signal peptide of *Arabidopsis* WAK2 at the N-terminus of mCherry and the ER retention signal His-Asp-Glu-Leu at the C-terminus. The fluorescence images were captured using a 60× water-immersion lens, with a 488 nm laser for GFP fusion excitation and a 534 nm laser for mCherry excitation using a multiphoton laser scanning microscope (Olympus FV1000 MPE).

### Yeast expression and *in vivo* characterization

The coding regions of each isolated gene were subcloned into the pYeDP60 yeast expression vector downstream of the *GAL1* promoter. All constructs, including the empty vector as a negative control, were transformed into the WAT11 yeast strain using the lithium acetate method according to the manufacturer’s instructions (Clontech, Japan). *In vivo* yeast assays were performed, as previously described, with modifications^19^. The cultures were extracted with an equal volume ethyl acetate and evaporated under nitrogen gas. The residues were then resolved in 200 μL 80% methanol for further HPLC analysis.

### Site-directed mutagenesis and *in vitro* enzyme assays and quantification of FNSII proteins

Each site-directed mutagenesis version of the target proteins was prepared as described according to the manual for the Fast Mutagenesis System (Transgen, China) and subcloned into the pYeDP60 vector. Microsomal proteins of yeast strain WAT11 expressing the LjFNSIIs, LmFNSIIs, and their site-directed mutant versions were prepared as described previously with minor amendments^18^. The *in vitro* enzyme assays were carried out as described previously^49^. The expected monomers were extracted with an equal volume ethyl acetate containing 0.003 mg/mL diosmetin as the internal standard for further HPLC analysis.

To optimize the reaction temperature and pH values, the activities of the microsomal proteins were assessed with the substrate naringenin at serial temperatures (25 to 70 °C) and pH values (5.0 to 8.0). The effect of divalent cations on enzyme activity was evaluated in reaction mixtures supplemented with 10 mM of MgCl_2_, MoCl_2_, CaCl_2_, MnCl_2_, NiCl_2,_ CoCl_2_, ZnCl_2_, CuCl_2_, or FeCl_2_. The apparent *K*_*m*_values for different flavanone substrates were determined by incubating 100 μg of recombinant enzyme with serial concentrations of flavanone (0.5, 1, 2, 2.5, 5, 10, 20, 40, 80, 160, and 400 μM). Each reaction product was quantified using a standard curve generated from known concentrations of the corresponding flavone. Apparent *K*_*m*_ and *V*_*max*_ values were calculated from Lineweaver-Burk plots using Hyper 32 software. This experiment was carried out three times independently and there were three technical replications used in each analysis to obtain the accurate apparent *K*_*m*_and *V*_*max*_ values.

### HPLC analysis of enzyme reaction products

Aliquots of the above extracts were analyzed on a Dionex system (Sunnyvale, USA) with a P680 HPLC pump and an ODS-80Ts QA C-18 column (150 × 4.6 mm, 5 μm i.d.; Tosoh, Japan). Eluent A was 10% aqueous formic acid; eluent B was 15% methanol in acetonitrile. The following elution gradient was used: 15% B at 0 min, 35% B at 5 min, 37% B at 15 min and then a return to 15% B in 5 min. 20 μL of the sample extract was injected with a flow rate at 0.8 mL/min and a constant column temperature maintained at 35 °C. Chromatograms were acquired at 350 nm and 280 nm for identifying products from the enzyme reactions. DAD data were recorded from 200 to 800 nm. The products were quantified using LU, AP, and DHF as standards, with diosmetin as the internal standard.

### Homology modeling of the protein structure of LjFNSII-1.1

To select suitable templates for modeling, searches were performed with the sequence of LjFNSII-1.1 and LjFNSII-2.1, respectively, at the Swiss Model website (http://swissmodel.expasy.org//SWISS-MODEL.html). Among the most similar proteins, CYP1A1 (4i8v.1.A) was selected as the template. A three-dimensional homology model of LjFNSII-1.1 was generated using Modeler 9.10^29^ and the three-dimensional structure of eriodictyol was created in ChemDraw 3D Ultra. The docking was performed using the AutoDock-Tool program and sixty simulations were run with AutoDock 4.0^50^. A heme molecule, as the cofactor, was also docked in the generated protein model with the lowest energy configuration.

### Over-expression construct and promoter GUS assays in Tobacco

The coding regions of *LjFNSII-1.1, LjFNSII-2.1*, and *LmFNSII-1.1* were cloned into the pCAMBIA 2300 binary vector (Cambia, Australia) under the control of the cauliflower mosaic virus 35S promoter. The promoter of *LmFNSII* was amplified by genome walking and then inserted into the pCAMBIA 1391 binary vector, in frame with GUS at the C-terminal end (Cambia). The constructs were then introduced into *Agrobacterium tumefaciens* strain GV3101 and co-cultured with leaf sections of sterile *N. Benthamiana*. The empty vectors were transformed at the same time and represented the controls. The presence of and the expression of the transgenic FNSII constructs were confirmed in the transgenic lines by PCR and RT-PCR, respectively, with the empty-plasmid transgenic plantlets as controls. GUS staining was performed using a standard protocol as described previously^51^.

### UPLC-MS/MS analysis of flavones from plants and enzyme reaction products

For analysis of flavone compounds from plant materials, leaves or flowers were harvested and lyophilized before extracting with methanol^52^. For acid hydrolysis, samples were treated an equal volume of 1 M HCl^53^. Analyses were performed using an ACQUITY ultra performance liquid chromatography system (UPLC I-class, Waters, USA) with an ACQUITY UPLC HSS C18 column (1.7 μm, 100 × 2.1 mm i. d.; Waters); the flow rate was 0.4 mL/min and operated at 35 °C. The mobile phase consisted of water: formic acid (999:1, v/v; eluent A) and acetonitrile (eluent B). 1 μL of the sample extract was injected, and the elution program was 0 min, 5% B; 6 min, 45% B; 7 min, 90% B, and isocratic with 5% B (7.1–10 min). Absorbance values at 350 nm by a UV detector were used for the quantification of flavones and luteolin was used as the standard. All samples were analyzed in triplicate.

To confirm the products of plant metabolites and the enzyme reactions, samples were analyzed via LC-MS/MS analysis. The UPLC separation conditions were the same as those described above. The ESI-MS/MS analysis performed using a Xevo TQ-MS mass spectrometer (Waters). The compounds were acquired in both positive-ion (PI) and negative-ion (NI) mode. The detection conditions were as follows: desolvation temperature: 400 °C; desolvation gas (N_2_) rate, 800 L/h; cone gas flow, 50 L/h; cone voltage and capillary voltage were 30 V and 3 kV, respectively, for PI, and −60 V and 2 kV, respectively, for NI. Mass spectra were recorded across an *m/z* range of 100–1000, and MassLynx version 4.1 was used for system control and data processing.

### Gene expression analyses

Leaves and flowers at different developmental stages were collected, and total RNA was isolated as described above. First-strand cDNA was reverse transcribed using a FastQuant RT Kit With gDNase (Tiangen, China) and qRT-PCR was performed with SYBR-Green using a Light Cycler R480 Real-Time PCR System (Roche, Switzerland). The relative quantification of each transcript was performed in triplicate as described previously^54^, and the actin gene from *L. japonica* (KU127582) was chosen as a reference gene.

All primers used in this study are listed in Supplementary Table S6.

## Additional Information

**How to cite this article**: Wu, J. *et al.* Flavone synthases from *Lonicera japonica* and *L. macranthoides* reveal differential flavone accumulation. *Sci. Rep.* **6**, 19245; doi: 10.1038/srep19245 (2016).

## Supplementary Material

Supplementary Information

## Figures and Tables

**Figure 1:**
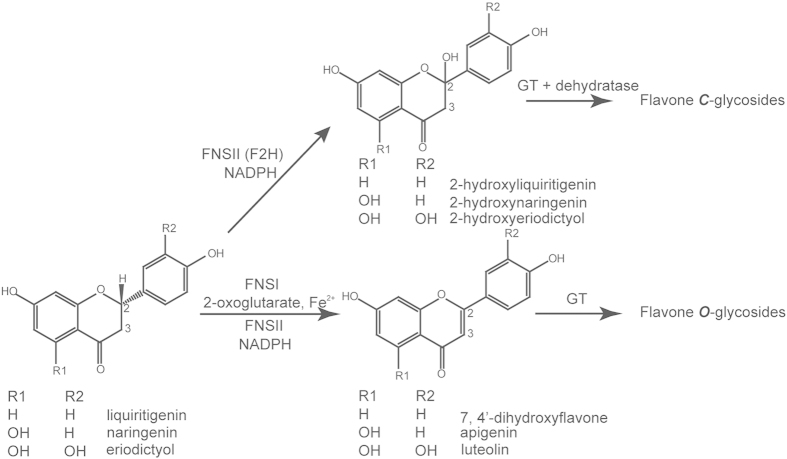
Biosynthesis pathway for flavones. There are two types of flavone synthase enzymes. The FNSI enzymes are soluble 2-oxoglutarate-dependent dioxygenases, and the FNSII enzymes are NADPH-dependent cytochrome P450 monoxygenases. Most of the FNSII enzymes display similar catalytic mechanisms as FNSI enzymes, converting flavanones to flavones directly, followed by different *O*-linked modifications. Some FNSII enzymes form a 2-hydroxylflavanone intermediate that serves as the substrate for *C*-glycosyl transferases that produce *C*-linked glucosides.

**Table 1:** LjFNSII-1.1, LjFNSII-2.1, and LmFNSII-1.1 activities under optimized assay conditions.

Substrate	Recombinants	*K*_*m*_ (μM)	*V*_*max*_ (nM s^−1^)	*K*_*cat*_ (s^−1^)	*K*_*cat*_*/K*_*m*_ (mM^−1^s^−1^)	Specific activity (nkat mg^−1^)
Eriodictyol	LjFNSII-1.1	5.07 (0.05)	4.45 (0.11)	2.56 (0.06)	504.38 (6.80)	22.27 (0.53)
	LjFNSII-2.1	2.05 (0.04)	1.64 (0.04)	0.94 (0.02)	459.53 (9.00)	8.20 (0.22)
	LmFNSII-1.1	3.09 (0.17)	0.77 (0.01)	0.44 (0.01)	142.50 (7.69)	3.83 (0.07)
Naringenin	LjFNSII-1.1	9.93 (0.64)	2.77 (0.18)	1.59 (0.10)	160.23 (6.16)	13.83 (0.88)
	LjFNSII-2.1	1.63 (0.06)	0.48 (0.02)	0.28 (0.01)	170.38 (5.45)	2.42 (0.12)
	LmFNSII-1.1	1.63 (0.12)	0.35 (0.02)	0.20 (0.01)	121.98 (5.26)	1.73 (0.11)
Liquiritigenin	LjFNSII-1.1	6.48 (0.26)	7.68 (0.23)	4.41 (0.13)	681.45 (14.86)	38.39 (1.15)
	LjFNSII-2.1	2.56 (0.03)	2.06 (0.00)	1.18 (0.00)	462.08 (4.41)	10.31 (0.02)
	LmFNSII-1.1	2.38 (0.07)	1.23 (0.04)	0.70 (0.10)	295.02 (3.60)	6.14 (0.20)

Data are expressed as means of triplicate experiments, and SE values are shown in parentheses.

**Table 2:** Comparison of the catalytic activities of the site-directed mutagenesis variants of the LjFNSII-1.1 and LmFNSII-1.1 enzymes using eriodictyol as the substrate.

Recombinants	Relative Activity (%)
LmFNSII-1.1	21.17 (1.08)
LmFNSII-1.1-V204M	222.68 (5.62)
LmFNSII-1.1-V379L	130.61 (13.01)
LjFNSII-1.1-M206V	13.57 (0.88)
LjFNSII-1.1-L381V	30.76 (1.13)
LmFNSII-1.1-A120S	0.35 (0.01)
LjFNSII-1.1-S122A	239.60 (39.62)
LjFNSII-1.1	100.00 (1.27)

Relative activity is expressed as a percentage of the activity measured for LjFNSII-1.1. The table shows mean ± SE of three independent experiments.
