# Supplementary material for: Flavone synthases from Lonicera japonica and L. macranthoides reveal differential flavone accumulation
Source: Sci Rep. 2016 Jan 12;6:19245. doi: 10.1038/srep19245 (PMC4709722; doi:10.1038/srep19245)
Supplement: Supplementary Information [file srep19245-s1.pdf]

Flavone synthases from *Lonicera japonica* and *L. macranthoides* reveal differential flavone accumulation

Jie Wu[1,2], Xiao-Chen Wang[2,3], Yang Liu[1,2], Hui Du[1], Qing-Yan Shu[1], Shang Su[1,2], Li-Jin Wang[1,2], Shan-Shan Li[1,*], Liang-Sheng Wang[1,*]

[1]Key Laboratory of Plant Resources/Beijing Botanical Garden, Institute of Botany, Chinese Academy of Sciences, Beijing 100093, China

[2]University of Chinese Academy of Sciences, Beijing 100049, China

[3]Key Laboratory of Plant Molecular Physiology, Institute of Botany, Chinese Academy of Sciences, Beijing 100093, China

* Authors for correspondence

LSW: wanglsh@ibcas.ac.cn

SSL: shshli@ibcas.ac.cn

Tel: +86 10 62836654

Fax: +86 10 62590348

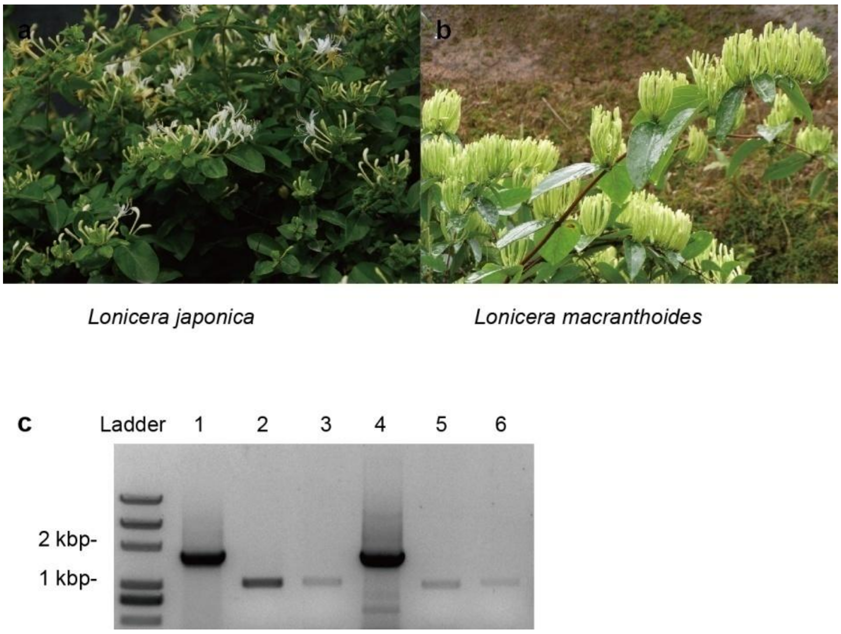

Supplementary Figure S1. The plant materials used in this study and the *FNSII* transcripts identified in this study. (a) *Lonicera japonica* Thunb. (b) *L. macranthoid*es Hand.-Mazz. (c) *FNSII* transcripts identified from *L. japonica* and *L. macranthoid*es. *FNSII* transcripts were cloned from cDNA of *L. japonica* and *L. macranthoides* by polymerase chain reaction assay. Lane 1, *LjFNSII-1.1&2.1*. The PCR products from lane 1 were cloned and sequenced, both *LjFNSII-1.1* and *LjFNSII-2.1* were recovered from the resulting clones; lane 2, *LjFNSII-1.2*; lane 3, *LjFNSII-2.2*; lane 4, *LmFNSII-1.1*; lane 5, *LmFNSII-1.2*; lane 6, *LmFNSII-2.2*, this sequence was cloned from *L. macranthoid*es and is identical with *LjFNSII-2.2*.

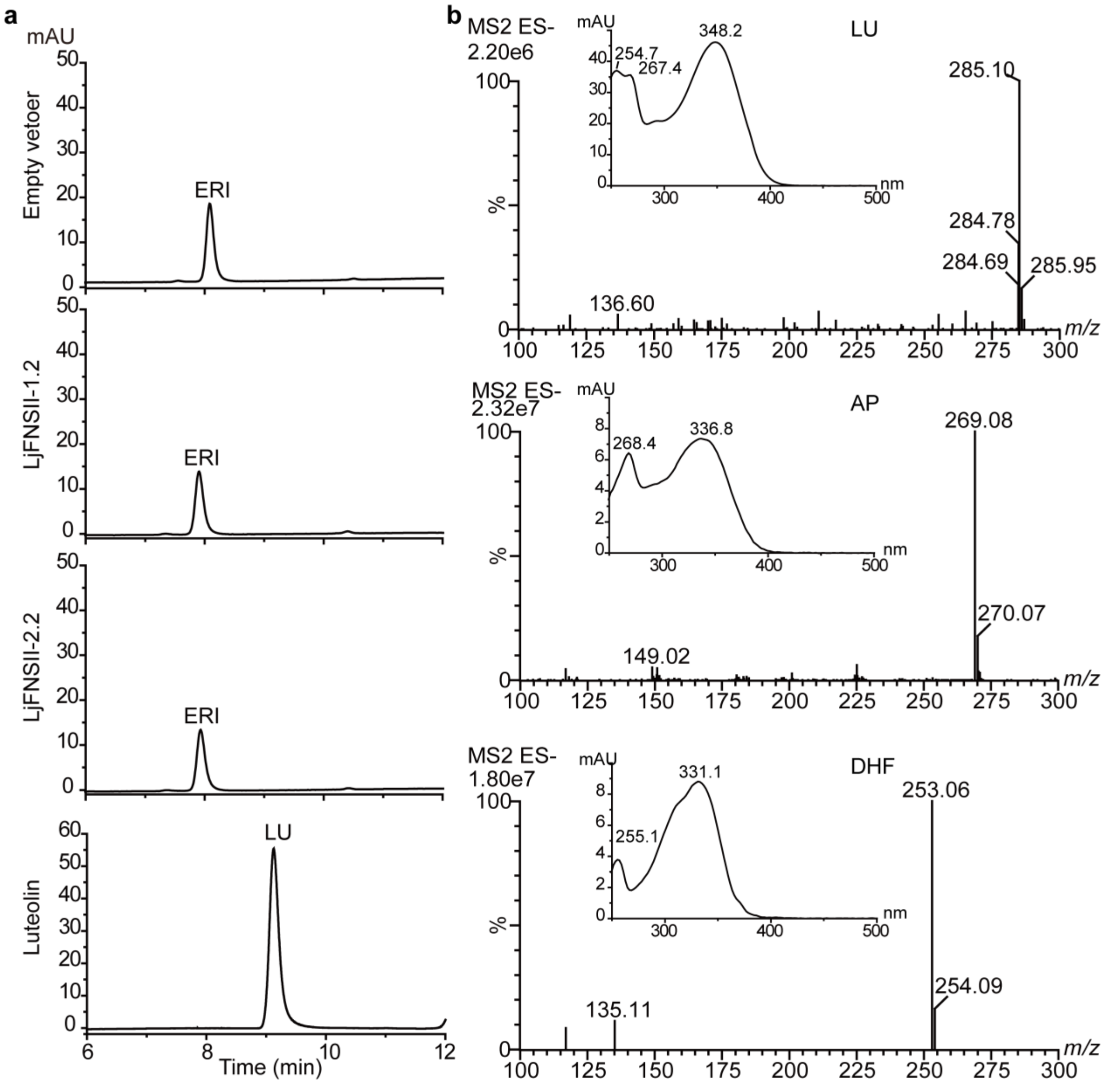

Supplementary Figure S2. HPLC profiles of extracts from yeast cells expressing short FNSII proteins and characterization of enzyme reaction products. (a) Yeast cultures fed with eriodictyol (ERI) as the substrate. HPLC chromatograms (350 nm UV spectra) showed no production of the expected luteolin (LU) from the yeast cells expressing LjFNSII-1.2, LjFNSII-2.2, and the empty vector. (b) Enzyme reaction products were characterized by comparison of UV absorption and mass spectrometric behavior with authentic standard compounds. The UV absorption and MS/MS fragmentation pattern of LU, apigenin (AP), and 7, 4'-dihydroxyflavone (DHF) were displayed above.

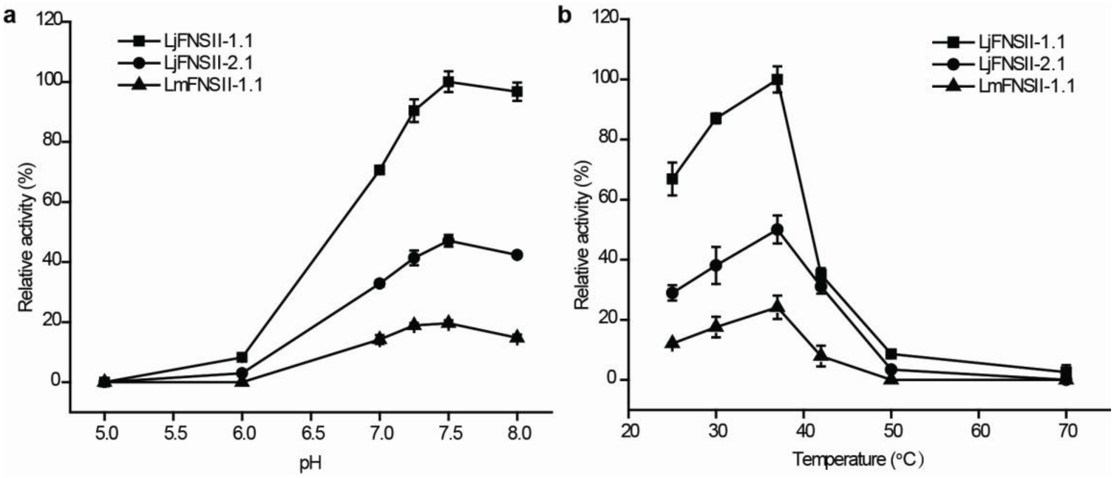

Supplementary Figure S3. Effects of pH and temperature on the enzyme activities of FNSII proteins. (a) Effects of pH on enzyme activities at 37℃ for 30 min. Relative activity are expressed as a percentage of the activity measured for LjFNSII-1.1 (100%) with conditions at pH 7.5. (b) Effects of temperature on enzyme activities at pH 7.5 for 30 min. Relative activity are expressed as a percentage of the activity measured for LjFNSII-1.1 (100%) at 37℃. The graph shows average values of three independent measurements with the respective error bars indicative of the standard deviation (SD).

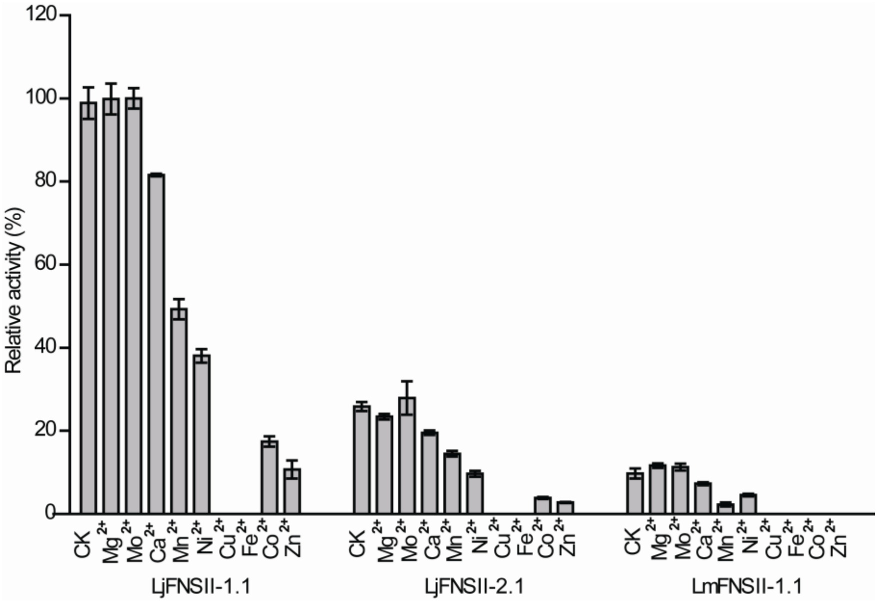

Supplementary Figure S4. Effects of various divalent metal ions on FNSIIs activities. Activity assays of FNSIIs were performed in reaction mixtures (pH 7.5) containing a series of 10 mM different metal ions (Mg^{2+}, Mo^{2+}, Ca^{2+}, Mn^{2+}, Ni^{2+}, Cu^{2+}, Fe^{2+}, Co^{2+}, Zn^{2+}) and incubated at 37℃ for 30 min. The potassium phosphate buffer without any extra metal ions was used as control check (CK). Relative activity of each recombinant protein under different reaction conditions are expressed as a percentage of the activity measured for LjFNSII-1.1 (100%) with 10 mM $MgCl_2$. The graph shows mean ± SD of three independent experiments.

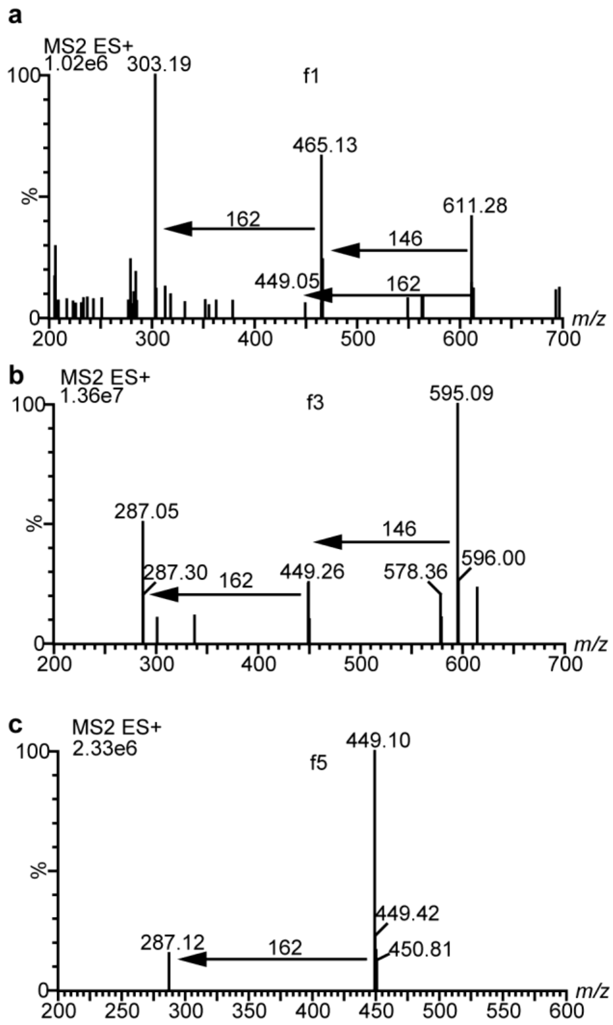

Supplementary Figure S5. The MS/MS fragmentation patterns of compounds from leaves of transgenic lines expressing LjFNSII-1.1. Peak f1 (a), peak f3 (b), and peak f5 (c) from leaves of transgenic *N. benthamiana* were identified according to their MS/MS fragmentation. Peak f1 had a diagnostic *m/z* 303 ion and successively lost 146 and 162 D in the parent compound (*m/z* 611 $[M+H]^+$). This peak was putatively assigned as quercetin-3-rhamnose-7-glucoside (Qu-3-Rha-7-Glc). Likewise, two additional peaks (f3 and f5) were putatively identified as luteolin-7-*O*-neohespeidoside (Lu-7-*O*-Neo) and keampferol-7-*O*-glucoside (Km-7-*O*-Glc). The three peaks were found to occasionally accumulate in leaves of some lines.

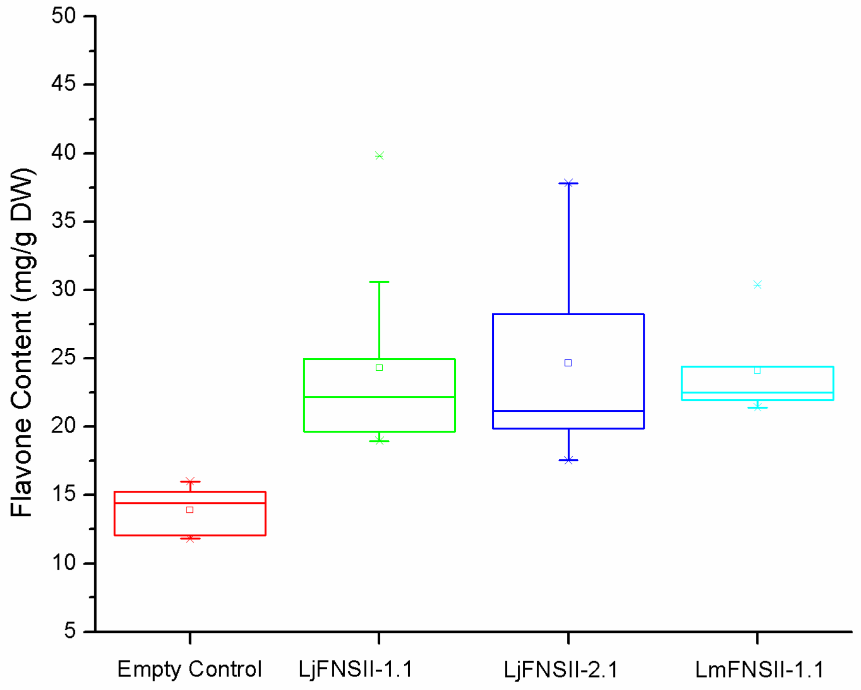

Supplementary Figure S6. **The total flavone contents in leaves of transgenic lines expressing LjFNSII-1.1, LjFNSII-2.1, and LmFNSII-1.1.** The total flavone contents of each transgenic line expressing empty vector, LjFNSII-1.1, LjFNSII-2.1, and LmFNSII-1.1 were measured. The graph shows mean ± SD of total flavones from more than three independent transgenic lines.

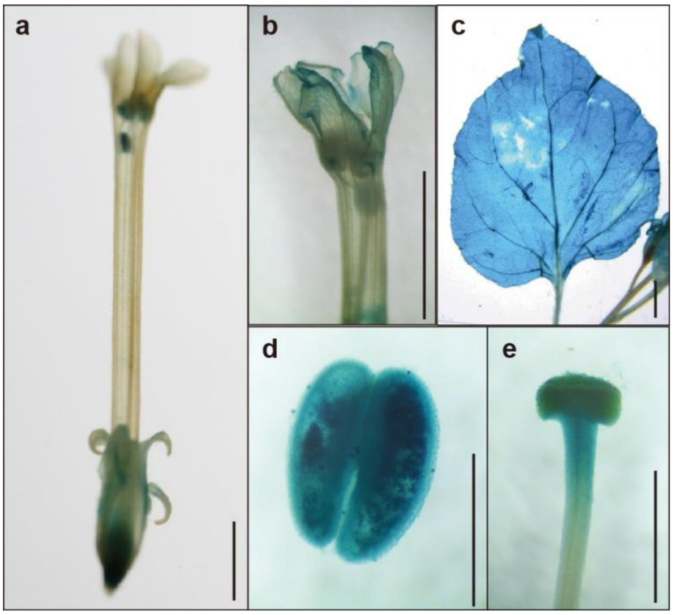

Supplementary Figure S7. The tissue-specific distribution of LmFNSII-1.1 promoter-GUS activities in transgenic *N. benthamiana*. GUS expression pattern of LmFNSII-1.1 promoter is very weak and hardly detected in flowers (a, b), while it is active throughout the leaf (c), anther (d) and stigma (e). Scale bars = 0.5 cm (a-c) and 1.0 mm (d, e).

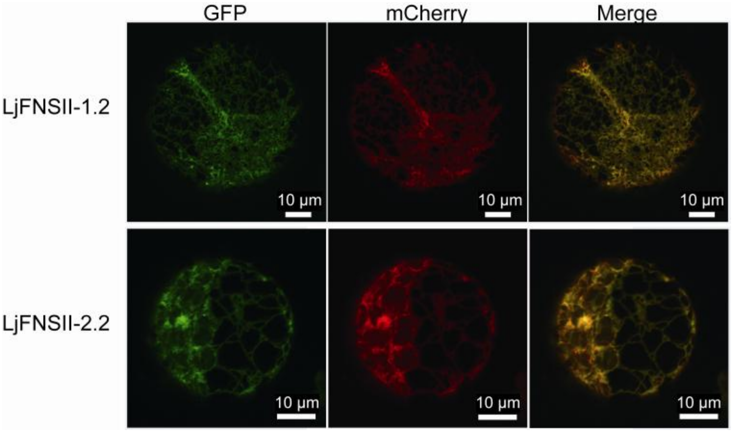

Supplementary Figure S8. The transient expression of FNSII-GFP fusion proteins in *Arabidopsis* protoplasts. Transient expression constructs (*35S-FNSII-GFP* and *35S-mCherry-HDEL*) were co-transformed into *Arabidopsis* protoplasts. Merge and overlay of FNSII-GFP fluorescence and mCherry-HDEL fluorescence (endoplasmic reticulum [ER] marker). Scale bars = 10 μm.

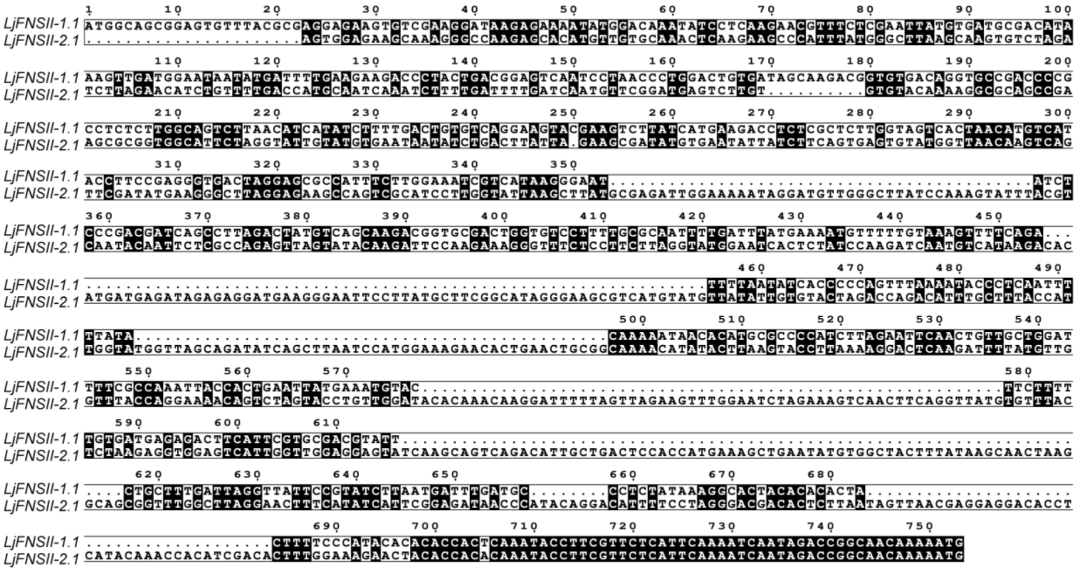

Supplementary Figure S9. Comparison of the 5' upstream regions of the *LjFNSII-1.1* and *LjFNSII-2.1* genes. Identical nucleotides are shown on a black background, and gaps are indicated by dashes.

Supplementary Table S1. Polypeptide sequences of FNSII proteins and actin identified from *L. japonica* and *L. macranthoides* in this study

Protein	Accession No.	Amino acid sequences
LjFNSII-1.1	KU127576	MWIFDLTISFTTLLFLIFTTALLLLLKVFKKNHKLRPPPSPFTLPIIGHLHLLGPLIHQSFHRLSTL<br>YGPLIQLKIGYIPCVVASTPELAKEFLKTHELAFSSRKHSAAIKLLTYDVSFAFSPYGPYWKFIK<br>KTCTFELLGTRNMNHFLPIRTNEIRRFLQVMLEKAKASEGVNVTEELIKLTNNVISQMMFSTR<br>SSGTEGEAEEMRTLVREVTQIFGEFNVSDFIKLCKNIDIGGFKKRSKDIQKRYDALLEKIISERE<br>SERARRGKNRETLGEEGGKDFLDMMLDTMEDGKCEVEITRDHIKALVLDFLTAATDTTAIAV<br>EWTLAELISNPEVFDKAREEIDKVVGKHRLVTELDTPNLPYIHAIIKESFRLHPPIPLLIRKSVQD<br>CTVGGYHISANTILFVNIWAIGRNPKYWESPMKFWPERFLESNGPGPVGSMDIKGHHYELLPF<br>GSGRRGCPGMALAMQELPVVLAAMIQCFNWKPVTLDGEELDMSERPGLTAPRAHDLVCVPS<br>ARINSFDNF
LjFNSII-2.1	KU127578	MWIFDLTISFTTLLFLIFTTALLLLLKVFKKNHKLRPPPSPFTLPIIGHLHLLGPLIHQSFHRLSTL<br>YGPLIQLKIGYIPCVVASTPELAKEFLKTHELAFSSRKHSAAIKLLTYDVSFAFSPYGPYWKFIK<br>KTCTFELLGTRNMNHFLPIRTNEIRRFLQVMLEKAKASEGVNVTEELIKLTNNVISQMMFSTR<br>SSGTEGEAEEMRTLVREVTQIFGEFNVSDFIKLCKNIDIGGFKKRSEDIQKRYDALLEKIISERES<br>ERARRGKNRETLGEEGGKDFLDMMLDTMEDGKCEVEITRDHIKALVLDFLTAATDTTAIAVE

		WTLAELISNPEVFDKAREEIDKVVGKHRLVTELDTPNLPYIHAIIKESFRLHPPIPLLIRKSVQD<br>CTVGGYHISANTILFVNIWAIGRNPKYWESPMKFWPERFLESNGPGPVGSMDIKGHHYELLPF<br>GSGRRGCPGMALAMQELPVVLAAMIQCFNWKPVTLDGEELDMSERPGLTAPRAHDLVCVPS<br>ARINSFDNF
LmFNSII-1.1	KU127580	MLIFDLTISFTTLLFLIFTTALLLKVFKKNHKLQPPPSPFTLPIIGHLHLLGPLIHQSFHRLSTLYG<br>PLIQLKIGYIPCVVASTPELAKEFLKTHELAFSSRKHSAAIKLLTYDVSFAFAPYGPYWKFIKKT<br>CTFELLGTRNMNHFLPIRTNEIRRFLQVMLEKAKASEGVNVTEELIKLTNNVISQMMFSTRSS<br>GTEGEAEEVRTLVREVTQIFGEFNVSDFIKLCKNIDIGGFKKRSEDIQKRYDALLEKIISERESER<br>ARRGKNRETLGEEGGKDFLDMMLDTMEDGKCEVEITRDHIKALVLDFLTAATDTTAIAVEWT<br>LAELISNPEVFDKAREEIDKVVGKHRLVTELDTPNLPYIHAIIKESFRLHPPIPLVIRKSVQDCTV<br>GGYHISANTILFINIWAIGRNPKYWESPMKFWPERFLESNEPGSVGSTDIKGHHYELLPFGSGR<br>RGCPGMALAMQELPVVLAAMIQCFNWKPVTLDGEELDMSERPGLTAPRAHDLVCVPSARIN<br>SFDNF
LjFNSII-1.2	KU127577	MWIFDLTISFTTLLFLIFTTALLLLLKVFKKNHKLRPPPSPFTLPIIGHLHLLGPLIHQSFHRLSTL<br>YGPLIQLKIGYIPCVVASTPELAKEFLKTHELAFSSRKHSAAIKLLTYDVSFAFSPYGPYWKFIK<br>KTCTFELLGTRNMNHFLPIRTNEIRRFLQVMLEKAKASEGVNVTEELIKLTNNVISQMMFSTR<br>SSGTEGEAEEMRTLVREVTQIFGEFNVSDFIKLCKNIDIGGFKKRSKDIQKRYDALLEKIISERE

		SERARRGKNRETLGEEGGKDFLDMMLDTMEDGKCEVEITRDHIKALVLVCTYFIILP
LjFNSII-2.2	KU127579	MWIFDLTISFTTLLFLIFTTALLLLLKVFKKNHKLRPPPSPFTLPIIGHLHLLGPLIHQSFHRLSTL<br>YGPLIQLKIGYIPCVVASTPELAKEFLKTHELAFSSRKHSAAIKLLTYDVSFAFSPYGPYWKFIK<br>KTCTFELLGTRNMNHFLPIRTNEIRRFLQVMLEKAKASEGVNVTEELIKLTNNVISQMMFSTR<br>SSGTEGEAEEMRTLVREVTQIFGEFNVSDFIKLCKNIDIGGFKKRSEDIQKRYDALLEKIISERES<br>ERARRGKNRETLGEEGGKDFLDMMLDTMEDGKCEVEITRDHIKALVLKKKRNRIQKRRSTY<br>VEEK
LmFNSII-1.2	KU127581	MLIFDLTISFTTLLFLIFTTALLLKVFKKNHKLQPPPSPFTLPIIGHLHLLGPLIHQSFHRLSTLYG<br>PLIQLKIGYIPCVVASTPELAKEFLKTHELAFSSRKHSAAIKLLTYDVSFAFAPYGPYWKFIKKT<br>CTFELLGTRNMNHFLPIRTNEIRRFLQVMLEKAKASEGVNVTEELIKLTNNVISQMMFSTRSS<br>GTEGEAEEVRTLVREVTQIFGEFNVSDFIKLCKNIDIGGFKKRSEDIQKRYDALLEKIISERESER<br>ARRGKNRETLGEEGGKDFLDMMLDTMEDGKCEVEITRDHIKALVLVCTDFIILP
LjACTIN	KU127582	SNWDDMEKIWHHTFYNELRVAPEEHPVLLTEAPLNPKANREKMTQIMFETFNVPAMYVAIQA<br>VLSLYASGRTTGIVLDSGDGVSHTVPIYEGYALPHAILRLDLAGRDLTDALMKILTERGYMFTT<br>TAEREIVRDMKEKLAYVALDYEQELETAKSSSSVEKNYELPDGQVITIGAERFRCPEVLFQPSL<br>IGMEAAGIHETTYNSIMKCDVDIRKDLYGNIVLSGGSTMFPGIADRMSKEITALAPSSMKIK

Supplementary Table S2. Accession numbers of FNSII proteins used for phylogenetic analysis

Protein	Species	Accession No.	Protein	Species	Accession No.
CYP93B1	*Glycyrrhiza echinata*	AB001380	CYP93B10	*Medicago truncatula*	DQ354373
CYP93B2	*Gerbera hybrida*	AF156976	CYP93B11	*Medicago truncatula*	DQ335809
CYP93B3	*Antirrhinum majus*	AB028151	CYP93B13	*Gentiana triflora*	AB193314
CYP93B4	*Torenia hybrida*	AB028152	CYP93B15	*Malus* × *domestica*	EG631280
CYP93B5	*Callistephus chinensis*	AF188612	CYP93B17	*Lobelia erinus*	AB221081
CYP93B6	*Perilla frutescens* var. *crispa*	AB045592	CYP93B18	*Camellia sinensis*	FJ169499.1

Supplementary Table S3. The activities of site-directed mutatant versions of the LjFNSII-1.1, LjFNSII-2.1, and LmFNSII-1.1 proteins with eriodictyol as the substrate under optimized conditions

Polypeptide	K_m	V_{max}	K_{cat}	K_{cat}/K_m	Specific activity
	μM	nM s^{-1}	s^{-1}	mM^{-1} s^{-1}	nkat mg^{-1}
LmFNSII-1.1-E240K	2.89 (0.21)	1.11 (0.05)	0.64 (0.03)	222.00 (10.20)	5.57 (0.24)
LjFNSII-2.1-E242G	3.96 (0.22)	3.67 (0.18)	2.11 (0.11)	531.83 (2.63)	18.34 (0.92)
LmFNSII-1.1-V204M	8.47 (0.52)	8.87 (0.52)	5.08 (0.30)	599.53 (1.43)	44.37 (2.61)
LjFNSII-1.1-M206V	3.63 (0.5)	0.87 (0.04)	0.50 (0.02)	141.20 (11.47)	4.36 (0.22)
LmFNSII-1.1-V379L	10.69 (0.55)	5.68 (0.11)	3.25 (0.06)	304.96 (13.78)	28.38 (0.55)
LjFNSII-1.1-L381V	6.68 (0.46)	2.09 (0.13)	1.20 (0.08)	180.02 (4.00)	10.45 (0.67)
LmFNSII-1.1-A120S	2.60 (1.15)	0.062 (0.01)	0.04 (0.00)	19.83 (7.75)	0.31 (0.02)
LjFNSII-1.1-S122A	10.53 (1.18)	10.58 (0.87)	6.08 (0.5)	581.71 (20.53)	52.92 (4.37)

The experiment was carried out more than three times and data are expressed as mean ± SE.

Supplementary Table S4. Identification of products accumulated in transgenic tobacco

No.	Identification	t_R (min)	λ_{max} (nm)	ESI-PI MS^2 (*m/z*)	ESI-NI MS^2 (*m/z*)
f1	Qu-3-Rha-7-Glc	3.45	255.36, 354.36	303 (100), 449 (5.87), 465 (66.63), 611 (41.76)	301 (22.06), 609 (100)
f2	Lu-7-*O*-Glc	3.65	265.36, 348.36	287 (17.28), 449 (100)	285 (49.57), 447 (100)
f3	Lu-7-*O*-Neo	3.76	265.36, 347.36	287 (50.67), 449 (25.48), 595 (100)	285 (11.39), 593 (100)
f4	Ap-7-*O*-Glc	4.09	266.36, 337.36	271 (27.39), 433 (100)	269 (12.74), 431 (100)
f5	Km-7-*O*-Glc	4.29	268.36, 341.36	287 (15.46), 449 (100)	285 (100), 447 (15.22)

Supplementary Table S5. The total flavone content in leaves from *Lonicera japonica* and *L. macranthoides*

	Total flavones (μg/g)
L. japonica	1843.74 ± 19.12
L. macranthoides	1037.16 ± 13.66

The total flavone content was measured as the average of three biological replicates. Data are expressed as mean ± SD

Supplementary Table S6. List of primers used in this study

Gene	Forward Primer (5'-3')	Reverse Primer (5'-3')	Purpose
*FNSII*s	GCCAAGGCTAGTGAGGGGGTGAACG	CTTGAGGAGGAGTAGGAGGGCGGTG	3' RACE and 5' RACE
	AGGGGGAGGCGGAGGAGATGAGGAC	GGGTGGAGAGGCGATGGAAGGACTG	
LjFNSII-1.1&2.1, *LmFNSII-1.1*	ATGTGGATCTTTGACCTC	AATACGCATACTCGTTGAAT	RT-PCR and genomic PCR
LjFNSII-1.2&2.2	TCTAGAATGTGGATCTTTGACCTC	CCATGGTTTCTCCTCCACGTAGGTGC	Cloning for Sub-cellular Localization
LjFNSII-1.1&2.1, *LmFNSII-1.1*	TCTAGAATGTGGATCTTTGACCTC	CCATGGAAAATTATCGAAAG	
LjFNSII-1.2&2.2	GGATCCATGTGGATCTTTGACCTC	GGTACCTCATTTCTCCTCCACGTAGGTG	Cloning for yeast expression
LjFNSII-1.1&2.1, *LmFNSII-1.1*	GGATCCATGTGGATCTTTGACCTC	GGTACCTTAAAAATTATCGAAAG	
LmFNSII-1-A120S	TGTATCATTTGCTTTTTCACCCTACGGT	AAAAAGCAAATGATACATCGTAGGTGA	Construction of

LjFNSII-1-S122A	TGTATCATTTGCTTTTGCACCCTACGGT	CAAAAGCAAATGATACATCGTAGGTGA	site-directed mutations
LmFNSII-1.1-V204M	AGGGGGAGGCGGAGGAGATGAGGAC	TCTCCTCCGCCTCCCCCTCGGTCCCCGA	
LjFNSII-1.1-M206V	AGGGGGAGGCGGAGGAGGTGAGGAC	CCTCCTCCGCCTCCCCCTCGGTCCCCGA	
LmFNSII-1.1-E240K	GGTTTAAGAAGAGAAGTAAGGATATA	TACTTCTCTTCTTAAACCCTCCAATATCA	
LjFNSII-2.1-E242G	GGTTTAAGAAGAGAAGTGGGGATATA	CCACTTCTCTTCTTAAACCCTCCAATAT	
LmFNSII-1.1-V379L	ACCCGCCAATTCCTCTGGTCATAAGAA	CCAGAGGAATTGGCGGGTGAAGCCGAA	
LjFNSII-1.1-L381V	ACCCGCCAATTCCTCTGCTCATAAGAA	GCAGAGGAATTGGCGGGTGAAGCCGAA	
LjFNSII-1.2&2.2	GGTACCATGTGGATCTTTGACCTC	TCTAGATCATTTCTCCTCCACGTAGGTG	over-expression in planta
LjFNSII-1.1&2.1, *LmFNSII-1.1*	GGTACCATGTGGATCTTTGACCTC	TCTAGATTAAAAATTATCGAAAG	
*FNSII*s	GATGCTTGATACTATGGAGGATGGC	TAGAATGAACCGAAACCGGCGGT	Genomic PCR for transgenic plant
*FNSII*s	TCATAGATGGCTGGAACAGAACCTC	CCCTAAAGCCAACAGAGAGAAGATG	RT-PCR for transgenic plant

LjFNSII-1.1&2.1, LmFNSII-1.1	CGCCATGATACAATGCTTTAA	ATCTCAACACCCACTTAAATAGG	RT-PCR
LjActin	CATTCCGATCAAAGAAGGCT	CTTGAAACTGCGAAGAGCAG	RT-PCR
NtActin	GCCAACAGAGAGAAAATGACCC	TCATGGATGGCTGGAAGAGGACTTC	RT-PCR
